# Incidental CT Findings in the Elderly with Low-Energy Falls: Prevalence and Implications

**DOI:** 10.3390/diagnostics12020354

**Published:** 2022-01-30

**Authors:** Sandra Niedermeier, Rebecca Wania, Alina Lampart, Robert Stahl, Christoph Trumm, Christian Kammerlander, Wolfgang Böcker, Christian H. Nickel, Roland Bingisser, Marco Armbruster, Vera Pedersen

**Affiliations:** 1 Department of General, Trauma and Reconstructive Surgery, University Hospital, LMU Munich, Marchioninistr. 15, 81377 Munich, Germany; sandra.niedermeier@outlook.de (S.N.); rebecca.wania@med.uni-muenchen.de (R.W.); christian.kammerlander@auva.at (C.K.); wolfgang.boecker@med.uni-muenchen.de (W.B.); 2 Department of Medicine, Kantonsspital Lucerne, Spitalstrasse, 6000 Lucerne, Switzerland; alina.lampart@luks.ch; 3 Institute of Diagnostic and Interventional Neuroradiology, University Hospital, LMU Munich, Marchioninistr. 15, 81377 Munich, Germany; robert.stahl@med.uni-muenchen.de (R.S.); christoph.trumm@med.uni-muenchen.de (C.T.); 4 Trauma Hospital Styria, Goestinger Straße 24, 8020 Graz, Austria; 5 Department of Emergency Medicine, University Hospital Basel, Petersgraben 2, 4031 Basel, Switzerland; christian.nickel@usb.ch (C.H.N.); roland.bingisser@usb.ch (R.B.); 6 Department of Radiology, University Hospital, LMU Munich, Marchioninistr. 15, 81377 Munich, Germany; marco.armbruster@med.uni-muenchen.de

**Keywords:** incidental findings, older adult, low-energy fall, emergency imaging, computed tomography

## Abstract

Background: Computed tomography (CT) is commonly used in trauma care, with increasing implementation during the emergency work-up of elderly patients with low-energy falls (LEF). The prevalence of incidental findings (IFs) resulting from CT imaging and requiring down-stream actions in this patient cohort is unknown. We have investigated the prevalence and urgency of IFs from emergency CT examinations in these patients. Methods: A total of 2871 patients with LEF and emergency CT examinations were consecutively included in this retrospective cohort study. The primary endpoint was the prevalence of IFs; the secondary endpoint was their urgency. Results: The median age was 82 years (64.2% were women). IFs were identified in 73.9% of patients, with an average of 1.6 IFs per patient. Of all IFs, 16.4% were classified as urgent or relevant, predominantly in the abdomen, chest and neck. Increasing age was associated with the prevalence of an IF (odds ratio: 1.053, 95% confidence interval: 1.042–1.064). Significantly more IFs were found in female patients (75.2% vs. 71.5%). Conclusion: IFs resulting from CT examinations of the elderly are frequent, but in more than 8 out of 10, they are harmless or currently asymptomatic. For the benefit of an accurate diagnosis of traumatic lesions, concerns about IFs with respect to disease burden, further work-up and resource utilisation might be disregarded.

## 1. Introduction

Computed tomography (CT) is a commonly used imaging modality in trauma care. Especially in patients suffering from high-energy trauma, whole-body CT (WBCT) scans are increasingly used and recommended by guidelines due to their real-time detection of acute traumatic injuries (ATI) with high specificity and sensitivity and their widespread availability [1,2,3,4]. However, besides their associated radiation exposure [1,2], WBCT scans are likely to reveal incidental findings (IF) unrelated to the preceding trauma [5,6,7,8,9,10,11,12,13,14,15,16,17]. Incidental findings are defined as recently unknown abnormalities revealed unintentionally in medical examinations. Their severity varies from harmless findings to ones requiring urgent treatment and follow-up. Several studies have reported on the prevalence and impact of IFs in predominantly severely injured trauma patients who received WBCT or selective CT scans as initial emergency imaging modality [5,6,8,9,10,18]. The prevalence of IFs in different trauma patient populations varies from 30.4% to 75.3% [5,6,8,9,10,12,13,14,15,16,18,19,20,21], with more findings detected by means of WBCT than by selective CT scans [10]. An amount of 1% to 46.9% of IFs could have an impact on patients’ health and require urgent treatment or further examination [5,10,11,17]. Interestingly, in chest CT performed on trauma patients, IFs are much more common than ATI; known relevant diagnoses, histories of smoking, and age serve as predicting factors for IFs [22].

Accurate and efficient emergency imaging of older adults presenting to the emergency department (ED) due to low-energy falls (LEF) is of increasing importance considering the general demographic development [23,24]. Due to certain limitations of the diagnostic accuracy of plain radiography, particularly in the thorax, spine and pelvic region [25,26,27], selective CT or unenhanced WBCT scans are frequently applied sequentially for diagnostic assurance [26]. Therefore, first-line WBCT or selective CT scans of selected older adults with LEF become increasingly important in daily practice in the emergency imaging setting. However, weighing the benefits of timely and accurate diagnosis of injuries against the disadvantages or harm of radiation exposure and IFs requiring downstream examinations [28] is obligatory for both emergency physicians and radiologists. So far, to the best of our knowledge, neither the distribution of IFs revealed by emergency CT scans nor their importance and relation to age and sex in older adults with LEF has been investigated systematically.

The objectives of this study were to assess, firstly, the prevalence of incidental CT findings in different body regions and, secondly, their urgency, regional distribution and relation to age and sex in a large cohort of elderly patients presenting with LEF to the ED.

## 2. Materials and Methods

### 2.1. Study Design

This is a secondary analysis of a bicentric, binational retrospective study carried out in two university tertiary care hospitals in Switzerland (University Hospital Basel) and Germany (University Hospital of Ludwig-Maximilians-University Munich) using electronic health records (EHRs). The study is in accordance with the declaration of Helsinki and was conducted using STROBE guidelines. Ethics approval was obtained from local ethics committees (EKNZ 2017-01078 approved 12 July 2017, EK LMU 17-217, approved 10 May 2017).

### 2.2. Study Population

Parts of the methods used in this study have been previously described [26]. In short, patients aged 65 years and older who suffered from LEF (falls from standing height, falls out of bed/from chairs/wheelchairs or other low-level furniture or falls from a low level less than 1 m [26]) and underwent CT examination of the head, cervical spine, chest, abdomen/pelvis and/or total body within 48 h of the index visit to the ED between 1 January 2016 and 31 December 2016 were consecutively included. Patients referred from another hospital with preceding imaging, patients who required trauma team activation, and patients with a delayed presentation (≥8 days after the fall) were excluded from this study.

### 2.3. Data Collection

Patients aged ≥65 years receiving a CT examination in one of the two hospitals within 48 h after admission in this 1-year-period were screened for inclusion using our radiology information systems (RIS) [26]. All EHRs of included cases were screened for validated CT reports from board-certified radiologists within 48 h of the index visit. Each of the identified CT reports was reviewed for documented IFs by two of the three trained non-blinded reviewers independently (S.N., R.W. and V.P.). An IF was defined as any finding not related to the trauma [20], independent of whether this finding might affect the patient’s health or not. When available, prior reports were checked to ensure the findings were new. Furthermore, EHRs were searched for delayed reported IFs during follow-up CT examinations. In the case of documented IFs, EHRs were searched for downstream examinations during the index admissions. Board-certified radiologists’ recommendations for additional imaging examinations were documented. Disagreements or equivocalness about the IFs and the categorisation of the IFs were decided upon by a third observer (V.P. and S.N.) by reviewing the CT images. Screening and chart review abstraction were conducted in accordance with the recommendations for medical chart review [29,30], which were fulfilled for 11 of 12 guidelines (abstractors were not blinded to the hypothesis). Data entry was performed in a Microsoft Access 2010/2016 database (Microsoft, Redmond, Washington, USA).

### 2.4. Incidental Findings and Categorisation

Incidental findings were categorised in accordance with a previously published study [8]. According to this, category 1 IFs were defined as findings with the need for urgent treatment or further examinations; category 2 IFs were defined as findings with the need for follow-up examinations within 3 to 6 months; category 3 IFs were defined as asymptomatic but potentially relevant in the future; and category 4 IFs were defined as harmless with no further investigation needed. The distinction between category 1 and 2 findings was made upon the board-certified radiologists’ recommendations for additional examinations and their scheduling in the CT reports and in the case of category 1 IFs documented down-stream treatments or examinations related to the findings (e.g., magnetic resonance tomography examinations, vascular intervention or diuretic treatment or drainage therapy of lung oedema or pleural effusion) during the index visit. Additionally, current guidelines and classification systems were applied for pulmonary nodules and renal cysts [31,32]. The default of the database entry template was designed considering the most common findings of the analysed body regions (head, neck, chest, abdomen including the pelvic region and spine) published previously [7], expecting comparable IFs in our cohort. Other IFs that were not listed were specified and categorised separately.

### 2.5. Key Outcome Measures

The primary endpoint of the study was to assess the prevalence of IFs in emergency CT imaging of older adults with LEF. The secondary outcomes were to determine the most common findings, their regional distribution, their urgency, and their relation to age and sex.

### 2.6. Statistics

For descriptive statistics, median and interquartile ranges (IQRs) were used to report continuous and ordinal data, where applicable. The Pearson Chi^2^ test with continuity correction or the Fisher’s exact test was used for the comparison of categorical data, with Bonferroni correction for multiple comparisons. Interrater agreement between reviewers was determined by calculating unweighted Cohen’s κ coefficients in a subsample of 868 patients for identification of IFs on CT reports using a corresponding 95% confidence interval (95% CI). All identified statistically significant risk factors (age, age category, sex) were chosen as covariates for the subsequent regression. For the outcome, IF multivariate logistic regression models were calculated and adjusted for age and sex. *p* values < 0.05 were considered significant. Statistical analyses were performed using SPSS Statistics 26 and RStudio version 1.4.1.

## 3. Results

**Figure 1:**
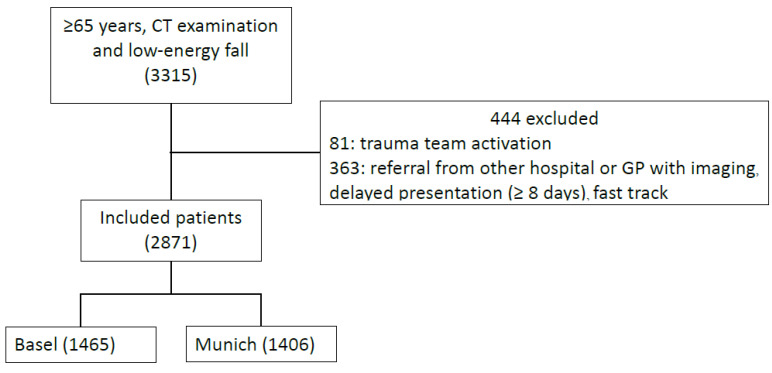
Inclusion and exclusion flow diagram of patient selection from 1 January 2016 to 31 December 2016 in Basel and Munich, with patients receiving computed tomography (CT) examinations of the head, spine, chest, abdomen, pelvic ring or proximal long bones during emergency department presentation or within 48 h.

We included 2871 patients in the analysis (Figure 1). The median age was 82 years (range 65–105; IQR 76–88), and 64.2% of included patients were women. Table 1 shows baseline demographic information. Cohen’s unweighted κ for the interrater agreement was 0.83 (95% CI: 0.79–0.87) for identification of IF on CT report. In total, 2122/2871 (73.9%) patients were identified with having IFs. The most frequent examinations were CT of the head (2549) and neck including the cervical spine (1614). CT examinations of the chest and abdomen (including the pelvic region) were performed in 262 and in 149 patients, respectively. Incidental findings in the thoracic and lumbar spine were registered in the CT scans of the selected spine regions or in the corresponding scans of the chest and abdomen.

Table 2 summarises the prevalence of IFs in the examined body regions. Overall, 3488 IFs in 2122 patients (on average, 1.6 IFs per patient) were found. Of these 3488 findings, 264 (7.6%) were classified as category 1, 307 (8.8%) as category 2, 2740 (78.5%) as category 3, and 177 (5.1%) as category 4 findings (Table 3).

**Figure 2:**
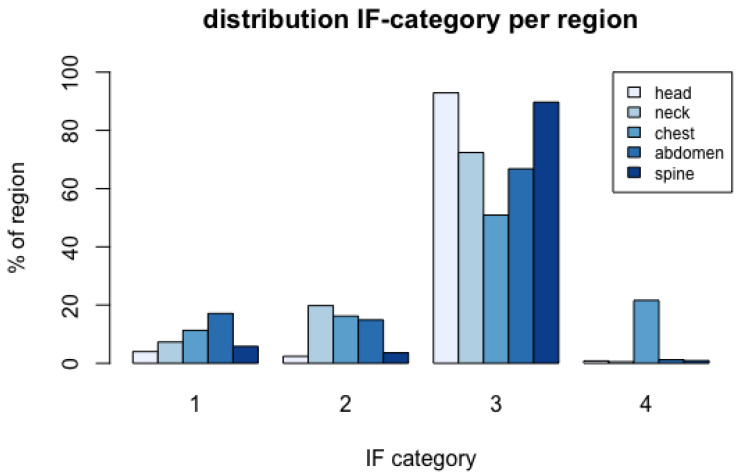
Distribution of incidental finding (IF) categories per body region investigated.

Figure 2 summarises the frequencies of IF categories per region. A detailed summary of the total numbers and proportions of IFs per category in the respective regions is given in Table S1. Category 1 IFs were most frequently present in the CT scans of the chest and abdomen; category 2 IFs were most frequently found in the neck and chest. Category 3 IFs were most frequently present in the head and spine. Increasing age is associated with the prevalence of an IF (OR: 1.053, 95% CI: 1.042–1.064, *p* < 0.001), and in the age group of ≥85 years, an IF was located in 82.7% of patients.

**Figure 3:**
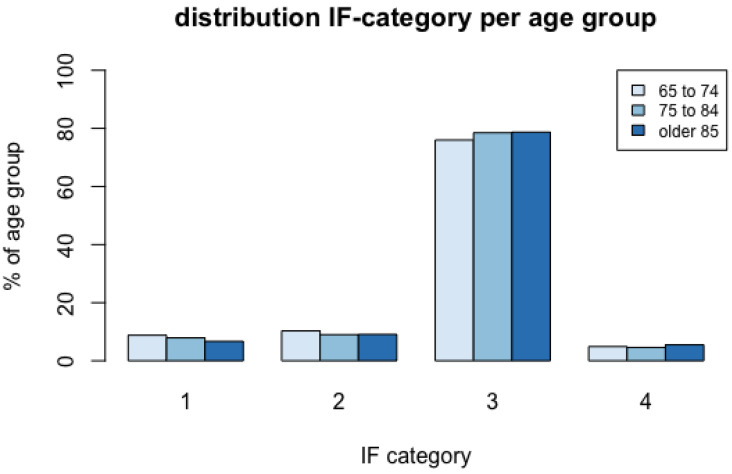
Distribution of incidental finding (IF) categories per age group.

Figure 3 summarises the proportions of IF categories by age group. A detailed summary of numbers and proportions of the severest IFs per region and age group is given in Table S2. Significantly more IFs of any category were found in female than male patients (75.2% vs. 71.5%) (Chi^2^: 4.73, df: 1, *p* = 0.03). There was no significant relation between age and sex and the severity of IFs in the head and the abdomen. In the neck region significantly more category 2 IFs were detected in female subjects (26.2% vs. 6.1%), and more category 3 IFs were detected in male subjects (86.1% vs. 65.5%; Chi^2^: 21.35, df: 3, *p* < 0.001). In the chest region, significantly more IFs of category 4 were detected in female subjects (22.9% vs. 11.4%; Chi^2^: 12.09, df: 3, *p* < 0.05). A detailed summary of numbers and proportions of the severest IFs per region and sex is given in Table S3. Significant relationships between age (Chi^2^: 22.45, df: 6, *p* = 0.001) and female sex (Chi^2^: 9.64, df: 3, *p* = 0.022) and IFs in the spine regions were measured. More category 3 IFs were detected in the oldest (93.8%) and female subjects (92.0% vs. 82.7%). Table 4 and Table 5 summarise the most frequent IFs per region and the most frequent category 1 and 2 findings per region.

## 4. Discussion

To the best of our knowledge, this is the first study examining the prevalence of IFs in older adults presenting to the ED with LEF and undergoing emergency CT scans for the detection of traumatic lesions. The main result of this study is that 73.9% of included patients had at least one IF in the examined body regions. Most IFs were seen in the abdomen, chest and head, and the vast majority of IFs detected were of minor impact, not requiring further diagnostics or treatment. Our data demonstrate age is a risk factor for IFs and that sex is related to IFs in certain body regions.

The overall IF prevalence of 73.9% in our study is confirmed by two other previously published studies in which 75% of patients undergoing WBCT scans showed IFs [5,8]. Several other authors [6,9,10,11,12,13,14,15,16,18,19,20,21] reported fewer occurrences of IFs, ranging from 15.9% [11] to 54.8% [16], regardless of whether WBCT or selective CT scans were conducted. A direct comparison of the prevalence of the above-mentioned studies is difficult due to varying patient inclusion criteria and general exclusion of certain diagnostic findings, such as degenerative joint diseases, age-related cerebral atrophy and atherosclerotic changes [6,7,10,12].

In line with previous studies [8,10,11,17,33], our analysis demonstrated that, besides the head, CT examinations of the abdomen and chest revealed the highest rates of IFs. This is presumably explainable by a large number of different visceral organs and tissues in the abdomen and chest.

Our evaluation indicated that 7.6% of IFs were identified as category 1, comprising patients requiring an urgent treatment or examination. This corresponds to previous results [6,7,8,9,10,11,15,16,20,21] reporting high urgency IFs in 2 [11] to 12.5% [7]. Most category 1 findings were found in the chest, followed by the abdomen and the head (see Figure 2), notably consisting of malignancies and pneumonia. Likewise, category 2 findings were located predominantly in the neck, chest and abdomen (see Figure 2). Lung nodules represent the majority of this severe category, followed by multinodular goitre and vascular abnormalities such as aortic elongations, ectasia and aneurysms. In total, 16.4% of IFs were categorised as urgent or relevant, demanding short-term treatment or follow-up investigations. Three considerations are relevant to the most common IFs in these categories. Firstly, the most common IFs concern findings that respond well to treatment, such as pneumonia and multinodular goitre, and thus could have a positive impact on patients’ lives. Secondly, it is possible that these findings will become symptomatic sooner or later, where later detection could worsen the outcome [34]. However, in the case of the very old, the benefit of this observation must be questioned because the diagnosis may not be life-limiting. Thirdly, since in most of the older adults with LEF, the origin of the falls remains unclear [23], some of the IFs may refer to the condition underlying the fall, e.g., an acute infection.

It should be noted that 83.6% of all IFs are category 3 and 4, thus currently asymptomatic or harmless. It can be assumed that these IFs with low impacts represent the average prevalence of certain age-related morbidities such as vascular diseases. Some of these may have already been diagnosed and treated so that no additional effort and resource utilisation is expected. In our own experience and consistent with other authors [6,7,8,10,12,13,14,17,34], a lack of systematic documentation and communication of IFs is evident, demanding digital solutions and general guidelines about communication of IFs [22]. The median age of our retrospective study cohort was 82 years; as a result, this analysis of IFs has the oldest trauma population published so far. Our data demonstrate that increasing age constitutes a risk factor for the detection of IFs in emergency CT imaging. This is confirmed by several previous studies in trauma [5,7,8,9,11,13,15,16,20,34,35] and mixed cohorts [17] with mean ages ranging from 36 [7] to 63 years [5]. Age was identified as an independent risk factor of IFs [17], not only in age groups but also in every year of increasing age [11,33]. Furthermore, a correlation between increasing age and severity of the IFs has been reported previously [8,9]. In our data, this could only be confirmed in spine CT examinations. Our in-depth analysis of IF severity revealed that category 3 IFs are more frequent in the oldest patients (85 years and older), whereas category 1 IFs (e.g., osteolysis, unclear masses) are more frequent in the youngest age group (65 to 74 years).

Our study adds to existing data regarding the relation between sex and IF category in certain body regions. According to this, female subjects have a higher risk of category 2 IFs in the neck, mainly multinodular goitre and thyroid lesions. The latter reflects the known higher prevalence in females of thyroid-associated diseases.

Based on this and previous studies, medico-economic impacts such as cost–benefit and medical benefit–burden ratios resulting from the detection of IFs in imaging studies remain unclear. It has been demonstrated that between 5.3% [17] and 6.2% [33] of all detected IFs generate additional investigations or clinical actions in their respective institutions. Based on this, an average cost of EUR 2292 per IF, which triggered down-stream actions, has been calculated in a mixed ED cohort [17]. However, with regard to all detected IFs, average costs would amount to EUR 121 per IF detected in this study. With regard to medical benefits or burdens, clear medical benefits have been determined for 1% of the cases, whereas clear medical burdens were determined for 0.5% of the cases, and in 4.6% of the instances, benefit–burden ratios were unclear [33]. It must be taken into account that an IF detected by a recent CT examination, which is clarified and documented systematically, would therefore no longer require cost-intensive clarifications in later stages. To address this properly, well-designed prospective cost–benefit and cost-effectiveness studies are needed.

Our study has several strengths, including a large consecutive sample of a representative population with rigorous chart review abstraction of key outcome measurements. On the other hand, the study is limited by its retrospective design without the systematic follow-up of patients and the initial patient selection representing a potential selection bias, as stated previously [26]. The selection of patients with unclear abdominal or thoracic complaints who received specific CT examinations may reveal a different pattern of IFs and severity. Furthermore, only selective CT scans were analysed. Thus, the prevalence of IFs can only be related to the examinations performed, resulting in a selection bias and a possible underestimation of the actual prevalence of IFs. This assumption is supported by a previous study where higher IF rates were found in WBCT compared to selective CT scans [10]. Additionally, since digital patient reports are not generally available, it is possible that our findings are pre-known diagnoses, which in some cases may result in over-reporting.

## 5. Conclusions

In conclusion, our study demonstrates that IFs revealed by emergency CT examinations in elderly adults are frequent, depicting increasing prevalence with increasing age. Of these, more than 8 out of 10 IFs are harmless or currently asymptomatic with potential impacts in the future and reflect the most common underlying age-related conditions such as vascular changes. According to our data, less than 2 out of 10 IFs require down-stream examinations or treatments. Considering the growing utilisation of emergency CT examinations in elderly adults with LEF, the concerns about IFs with respect to disease burden, necessary further work-up and resource utilisation might be disregarded when compared to the benefits of an accurate and prompt diagnosis of traumatic lesions.

## Figures and Tables

**Table 1:** Baseline characteristics of 2871 elderly adult patients presenting with low-energy falls from 1 January 2016 to 31 December 2016.

Characteristics	Total (*n* = 2871)	Basel (*n* = 1465)	Munich (*n* = 1406)
Age (median, IQR)	82 (76–88)	82 (70–94)	81 (68–94) ^b^
65–74 (%)	616 (21.5)	319 (21.8)	297 (21.1)
75–84 (%)	1146 (39.9)	555 (37.9)	591 (42.0)
>85 (%)	1109 (38.6)	591 (40.3)	518 (36.8) ^c^
Female (%)	1842 (64.2)	936 (63.9)	906 (64.4) ^a^

If not otherwise stated, data are reported as number of patients (%). ^a^ *p* = 0.76 (Pearson Chi^2^ test) between centres, ^b^ *p* = 0.39 (*t*-test) between centres, ^c^ *p* = 0.064 (Pearson Chi^2^ test) between centres and age categories. IQR: interquartile range.

**Table 2:** Summary of the prevalence of incidental findings per age group and per examination of different body regions (number of examinations).

Incidental Findings	Patients with An IF (%)
Overall	2122/2871 (73.9)
65 to 74 years	387/616 (62.8%)
75 to 84 years	818/1146 (71.4%)
≥85 years	917/1109 (82.7%)
	**Per CT Examination (%)**
Head CT (*n* = 2549)	1677/2549 (65.8)
Cervical spine CT (*n* = 1614)	179/1614 (11.1)
Chest CT chest (*n* = 262)	196/262 (74.8)
Abdomen CT (*n* = 149)	116/149 (77.9)
Neck CT (*n* = 1614)	346/1614 (21.4)

CT: computed tomography; IF: incidental finding.

**Table 3:** Proportion of incidental findings per category according to [8].

Category	Definition	%
1	Urgent treatment or further examination	7.6
2	Follow-up within 3 to 6 months	8.8
3	Asymptomatic but potentially relevant	78.5
4	Harmless, no further investigation	5.1

**Table 4:** Top 5 incidental findings per region.

Head (*n* = 1726)	Neck (*n* = 399)	Chest (*n* = 717)	Abdomen (*n* = 422)	Spine (*n* = 224)
Microangiopathy (1216) Previous cerebral infarction (310) Atherosclerosis (intracranial carotid artery, circle of Willis) (223) Lacunar lesions (124) Meningioma (65)	Atherosclerosis (extracranial carotid artery) (188) Multinodular goitre (100) Goitre (75) Regressive thyroid changes (70) Calcified thyroid nodule (13)	Atherosclerosis (aorta and branches) (254) Pleural scarring (173) Coronary artery calcification (137) Pleural effusion (118) Cardiomegaly (75)	Atherosclerosis (aorta and branches) (156) Diverticulosis (114) Kidney cysts (97) Liver cysts (40) Hiatal hernia (37)	Severe foraminal stenosis (60) Disc protrusion (20) Osseous lesion (9) Pars defect (9) Schmorl node (9)

**Table 5:** Top 5 incidental findings per region categorised 1 and 2.

Category	Head	Neck	Chest	Abdomen	Spine
1	*n* = 69	*n* = 29	*n* = 81	*n* = 72	*n* = 13
	Brain masses (31) Metastases/Osteolysis (10) Suspected normal pressure Hydrocephalus (8) Meningioma (6) Atherosclerosis (intracranial carotid artery, circle of Willis) (6)	Atherosclerosis (extracranial carotid artery) (8) Multinodular goitre (7) Mass (5) Lymphadenopathy (4) Hypodense thyroid lesion (2)	Infiltrates/Pneumonia (44) Lymphadenopathy (13) Lung nodules (12) Pleural effusion (11) Pulmonary oedema (8)	Mass/Metastases (13) Solid liver lesion of unclear aetiology (11) Adrenal myolipoma (10) Renal mass (9) Abdominal aortic aneurysm (6)	Osteolysis (8) Mass (4) Suspected Myelopathy (1)
**2**	***n*** **= 41**	***n*** **= 79**	***n*** **= 116**	***n*** **= 63**	***n*** **= 8**
	Meningioma (16) Suspected normal pressure hydrocephalus (12) Mass (9) Cerebral artery aneurysms (7) Atherosclerosis (intracranial carotid artery, circle of Willis) (2)	Multinodular goitre (34) Atherosclerosis (extracranial carotid artery) (17) Goitre (13) Hypodense thyroid lesions (10) Thyroid mass (6)	Lung nodules (47) Aortic lesions (18) Cardiomegaly (18) Aortic ectasia (14) Lymphadenopathy (8)	Abdominal aortic aneurysm < 4 cm (16) Prostate hyperplasia (8) Solid liver lesion (suspected for haemangioma) (7) Liver cyst (6) Hiatal hernia (4)	Severe foraminal stenosis (3) Distracted disc space (3) Haemangioma (1) Atypical haemangioma (1)

## Data Availability

Anonymized data presented in this study are available upon reasonable request from the corresponding author.

## Supplementary Materials

The following supporting information can be downloaded at https://www.mdpi.com/article/10.3390/diagnostics12020354/s1, Table S1: Summary of total numbers and proportions (%) of incidental findings (IFs) per category, Table S2: Summary of numbers and proportions (%) of severest incidental findings per region and age group, Table S3: Summary of numbers and proportions (%) of severest incidental findings per region and sex.
