# Supplementary material for: Incidental CT Findings in the Elderly with Low-Energy Falls: Prevalence and Implications
Source: Diagnostics (Basel). 2022 Jan 30;12(2):354. doi: 10.3390/diagnostics12020354 (PMC8871195; doi:10.3390/diagnostics12020354)
Supplement: Supplementary file 1 [file diagnostics-12-00354-s001.zip › diagnostics-1511707 SI.pdf]

Supplementary Materials

Supplementary Tables

Table S1. Summary of total numbers and proportions (%) of incidental findings (IF) per category.

		**1**	**2**	**3**	**4**
	**IF**	**IF (%)**	**IF (%)**	**IF (%)**	**IF (%)**
head	1726	69 (4,0)	41 (2,4)	1603 (92,9)	13 (0,8)
neck	399	29 (7,3)	79 (19,3)	289 (72,4)	2 (0,5)
chest	717	81 (11,3)	116 (16,2)	365 (50,9)	155 (21,6)
abdomen	422	72 (17,1)	63 (14,9)	282 (66,8)	5 (1,2)
spine	224	13 (5,8)	8 (3,6)	201 (89,7)	2 (0,9)
overall	3488	264 (7,6)	307 (8,8)	2740 (78,6)	177 (5,1)

Table S2. Summary of numbers and proportions (%) of severest incidental findings per region and per age group.

	**1**			**2**			**3**			**4**		
	**65-74**	**75-84**	**≥85**	**65-74**	**75-84**	**≥85**	**65-74**	**75-84**	**≥85**	**65-74**	**75-84**	**≥85**
head	15 (5,4)	28 (4,3)	28 (3,7)	10 (3,6)	23 (3,5)	25 (3,3)	251 (90,6)	599 (91,7)	7708 (92,9)	1 (0,4)	3 (0,5)	1 (0,1)
neck	4 (6,3)	13 (9,5)	12 (6,6)	14 (21,9)	22 (16,1)	41 (22,7)	46 (71,9)	101 (73,7)	127 (70,2)	0 (0)	1 (0,7)	1 (0,6)
chest	17 (15,7)	31 (15,0)	31 (12,0)	21 (19,4)	44 (21,3)	46 (17,8)	56 (51,9)	95 (45,9)	123 (47,7)	14 (13,0)	37 (17,9)	58 (22,5)
abdomen	14 (21,2)	24 (19,4)	34 (21,7)	12 (18,2)	22 (17,7)	22 (14,0)	39 (59,1)	78 (62,9)	99 (63,1)	1 (1,5)	0 (0)	2 (1,3)
spine	3 (7,1)	7 (8,0)	3 (3,1)	2 (7,1)	3 (2,3)	3 (3,1)	32 (76,2)	78 (89,7)	90 (93,8)	4 (9,5)	0 (0)	0 (0)
overall	53 (9,5)	103 (8,5)	108 (7,4)	60 (10,8)	113 (9,4)	137 (9,4)	424 (76,1)	951 (78,7)	1147 (78,9)	20 (3,6)	41 (3,4)	62 (4,3)

Table S3. Summary of numbers and proportions (%) of severest incidental findings per region and sex.

	1		2		3		4	
	**female**	**male**	**female**	**male**	**female**	**male**	**female**	**male**
head	44 (4,0)	27 (4,5)	39 (3,6)	19 (3,1)	999 (91,9)	559 (92,4)	5 (0,5)	0 (0)
neck	21 (7,9)	8 (7,0)	70 (26,2)	8 (6,1)	175 (65,5)	99 (86,1)	1 (0,4)	1 (0,9)
chest	47 (12,4)	32 (16,6)	70 (19,5)	35 (19,2)	167 (45,3)	102 (52,8)	86 (22,9)	22 (11,4)
abdomen	45 (19,0)	25 (24,5)	34 (14,8)	21 (19,1)	154 (65,0)	62 (56,4)	3 (1,3)	0 (0)
spine	8 (5,3)	5 (6,7)	4 (2,7)	4 (5,3)	138 (92,0)	62 (82,7)	0 (0)	4 (5,3)
